# Circular RNA circANKRD36 regulates Casz1 by targeting miR‐599 to prevent osteoarthritis chondrocyte apoptosis and inflammation

**DOI:** 10.1111/jcmm.15884

**Published:** 2020-11-17

**Authors:** Jian‐Lin Zhou, Shuang Deng, Hong‐Song Fang, Xian‐jin Du, Hao Peng, Qiong‐jie Hu

**Affiliations:** ^1^ Department of Orthopedics Renmin Hospital of Wuhan University Wuhan China; ^2^ Department of Emergency Renmin Hospital of Wuhan University Wuhan China; ^3^ Department of Radiology Tongji Hospital Tongji Medical College Huazhong University of Science and Technology Wuhan China

**Keywords:** Casz1, circANKRD36, miR‐599, osteoarthritis

## Abstract

Osteoarthritis (OA) is an ageing‐related disease characterized by articular cartilage degradation and joint inflammation. circRNA has been known to involve in the regulation of multiple inflammatory diseases including OA. However, the mechanism underlying how circRNA regulates OA remains to be elucidated. Here, we report circANKRD36 prevents OA chondrocyte apoptosis and inflammation by targeting miR‐599, which specifically degrades Casz1. We performed circRNA sequencing in normal and OA tissues and found the expression of circANKRD36 is decreased in OA tissues. circANKRD36 is also reduced in IL‐1β–treated human chondrocytes. FACS analysis and Western blot showed that the knockdown of circANKRD36 promotes the apoptosis and inflammation of chondrocytes in IL‐1β stress. We then found miR‐599 to be the target of circANKRD36 and correlate well with circANKRD36 both in vitro and in vivo. By database analysis and luciferase assay, Casz1 was found to be the direct target of miR‐599. Casz1 helps to prevent apoptosis and inflammation of chondrocytes in response to IL‐1β. In conclusion, our results proved circANKRD36 sponge miR‐599 to up‐regulate the expression of Casz1 and thus prevent apoptosis and inflammation in OA.

## 1 INTRODUCTION

OA is the most common degenerative joint disease affecting more than 25% of the population in adults.^1^, ^2^ It is also among the diseases with the fastest growing incidence owing to the ageing world population.^1^, ^2^, ^3^ However, there are still few interventions to decelerate the disease progression, because of the poor understanding about the molecular mechanisms underlying OA initiation and progression.^4^ Inflammation and chondrocyte cell death play prominent roles in OA progression.^1^, ^4^ Thus, the prevention of the chondrocyte cell death and inflammation may contribute to potential therapeutic strategy for OA treatment.

In recent years, microRNAs (miRNAs) have been found to play important roles in many inflammatory diseases including OA.^5^, ^6^ In addition, RNA sequencing (RNAseq) has identified multiple families of non‐coding RNAs, such as long intergenic non‐coding RNAs (lncRNA) and circular RNAs (circRNAs).^7^, ^8^ Compared with linear RNAs, circRNAs are more stable because of the non‐canonical splicing without a free 3′ or 5′ end.^9^ circRNA can function as miRNA sponges, whose sequences can competitively bind miRNAs to regulate the expression of target genes.^10^ Our investigations focused on cirRNA ankyrin repeat domain 36 (circANKRD36), which is markedly decreased in OA patients and has been reported to be related to inflammatory response in type 2 diabetes.^11^

Casz1 is an evolutionarily conserved zinc‐finger transcription factor originally characterized in *Drosophila*.^12^ Casz1 has critical functions in differentiation and tumour suppression.^13^, ^14^ Recently, it was reported that Casz1 is a regulatory protein controlling T‐helper cell inflammation and immunity.^15^

In this study, we demonstrated that circANKRD36 up‐regulates Casz1 by targeting miR‐599 to prevent chondrocyte apoptosis and inflammation in response to IL‐1β treatment. Our results suggested that the regulation of Casz1 by circANKRD36‐miR‐599 may present a novel strategy for the treatment of OA.

## 2 MATERIALS AND METHODS

### 2.1 Isolation and cultivation of human chondrocytes

Chondrocytes were enzymatically isolated from human cartilage of nine patients (mean age: 65, range: 51‐82 years). In short, full‐thickness cartilage was minced and digested for 45 min with 0.2% pronase (Sigma‐Aldrich), followed by washing and a second digest with 0.025% collagenase (Sigma‐Aldrich) overnight. After washing with PBS and filtration through a 40‐μm cell strainer, cells (passage 0) were cultured in serum‐containing medium. Chondrocytes were split at a confluence of 80% and used in passage 1 or 2.

### 2.2 Real‐time PCR analysis of mRNA

Total RNA was isolated with TRIzol reagent. The cDNA was synthesized from 2 μg of RNA by the Quantscript RT Kit (TiANGEN, KR103). The primer sequences of for RT‐PCR were as follows: circANKRD36, forward: GAGGCCACAAGTGATGAGA, reverse: CCTGGTGGTTTCTCAGAAGAC; miR‐599, forward: GUUGUGUCAGUUUAUCAAAC, reverse: CTCCATATCGCACTTTAATCTCTAACT; Casz1, forward: CAAAACAGACTCCATCACCACG, reverse: GTGCTGGCTGCCCGAGAAC; actin, forward: CCAACCGCGAGAAGATGA, reverse: CCAGAGGCGTACAGGGATAG; and U6, forward: CTCGCTTCGGCAGCACA, reverse: AACGCTTCACGAATTTGCGT.

### 2.3 Western blotting

In brief, the cells were harvested with a scraper and then washed once with cold PBS. The cells were then lysed in lysis buffer containing 50 Mm Tris‐HCl, 250 mmol/L NaCl, 5 mmol/L EDTA, 50 mmol/L NaF, 0.1% NP‐40 and 1% protease inhibitor cocktail. Equal amounts of proteins were size‐fractionated by 7.5‐15% SDS‐PAGE. Data collected came from at least three independent experiments.

### 2.4 Enzyme‐linked immunosorbent assay (ELISA)

Inflammatory cytokines, including IL‐6, IL‐8 and TNF‐α, in culture medium, were detected using ELISA Kits (R&D Systems, Minneapolis, MN, USA) according to the manufacturer's instructions.

### 2.5 Flow cytometry

Propidium iodide (PI) and FITC‐conjugated Annexin V staining (Beyotime) were used for flow cytometry. 1× PBS washed and PI/FITC‐Annexin V–stained cells in 50 μg/mL RNase A (Beyotime). After that, the cells were hatched (1 hour, dark, 25°C). Results were performed through fluorescence‐activated cell sorting (Beckman Coulter). Results were analysed by FlowJo software (Tree Star Software).

### 2.6 Reporter vectors constructs and luciferase reporter assay

The fragments of Casz1 (containing predicted binding sites) were cloned into the pMIR‐REPORT Vector (Promega) to form the reporter vector Casz1 wild‐type (Casz1‐wt). The sequence replacing the putative binding site was named Casz1 mutant (Casz1‐mut). The vector and the miR‐599 were cotransfected into HEK 293T cells to test the luciferase activity by Dual‐Luciferase Reporter Assay System (Promega).

### 2.7 Fluorescence in situ hybridization (FISH) analysis

Slides were treated with 0.2 mol/L HCl, washed for 5 minutes, incubated in 4% pepsase for 10 minutes and washed for 5 minutes. The slides were pre‐hybridized with pre‐hybridization for 2 hours, hybridization using probe was performed overnight at 37°C, and slides were rinsed with 0.3% NP‐40 for 30 minutes and counterstained with DAPI for 5 minutes. The images were acquired using a confocal microscope (Leica).

### 2.8 RNA pull‐down assay

RIP assay was conducted using Magna RIP Kit (EMD Millipore). Cells were lysed in RIP lysis buffer, and the cell lysate was treated with magnetic beads conjugated to human anti‐Ago2 antibody (Millipore) or control antibody (normal mouse IgG; Millipore). qRT‐PCR was performed to detect circANRKD36 expression.

### 2.9 CCK‐8 assay

Equal numbers of cells (approximately 5000/well) were seeded into a 96‐well plate 24 hours before experimentation. Cells were transfected with different plasmid or small RNA and then treated with IL‐1β for indicated time. After treatment, CCK‐8 was added into the 96‐well plate and incubated at 37°C for 1 hour. The absorbance of each sample was read at 450 nm.

### 2.10 Statistical analysis

For all statistical tests, three or more independent experiments were performed, and data are shown as means ± SD *P* < .05, by unpaired Student's *t* test, was considered statistically significant. Data were analysed by GraphPad 6.0 (Graph Pad Software).

### 2.11 Study approval

This study was approved by the Ethics Committee of Renmin Hospital of Wuhan University, Wuhan, China. Informed consent was obtained from each patient before the use of their cartilage tissue.

## 3 RESULTS

### 3.1 circANKRD36 is decreased in OA tissue and is inhibited by IL‐1β in human chondrocytes

**FIGURE 1:**
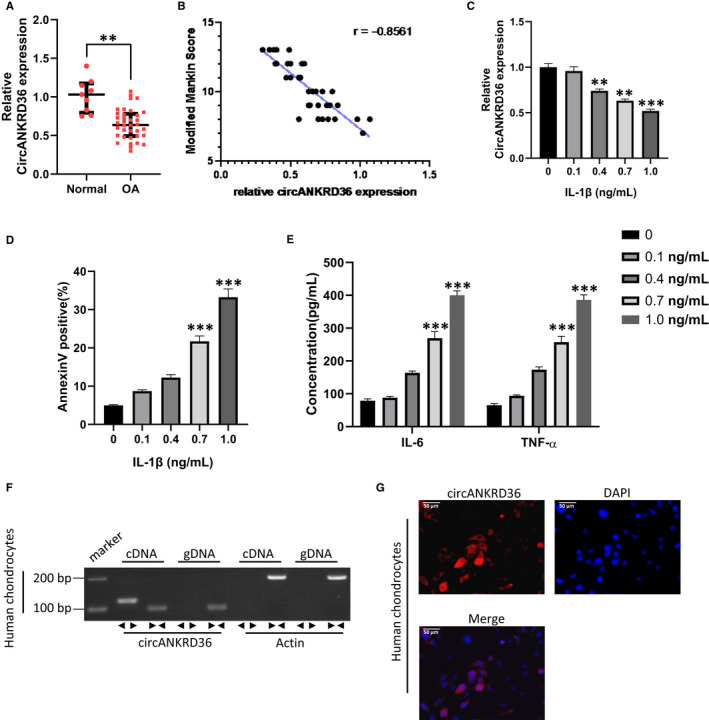
circANKRD36 is significantly decreased in osteoarthritis. A, Relative expression of circANKRD36 in OA (osteoarthritis) tissues (n = 36) and normal tissues (n = 9). **P < .01. B, Modified Mankin grading was performed to evaluate OA severity. (In the modified Mankin grading, abnormalities in structure (0‐6 points), cellularity (0‐3 points) and Safranin‐O staining (0‐4 points) were assessed up to a maximum score of 13 points.) The expression of circANKRD36 was negatively correlated with modified Mankin scores). C, Relative expression of circANKRD36 in chondrocytes treated with IL‐1β in a dose‐dependent manner (0, 0.1, 0.4, 0.7, 1.0 ng/mL). Data are presented as means ± SD (n = 3). **P < .01, ***P < .001. D, Human chondrocytes were treated with different doses of IL‐1β. Apoptotic cells were analysed by FACS with Annexin V staining. Annexin V–positive cell rates were statistically analysed. Data are presented as means ± SD (n = 3). **P < .01, ***P < .001. E, Human chondrocytes were treated with different doses of IL‐1β. Concentration of cytokines in culture medium was measured by ELISA Kit. Data are presented as means ± SD (n = 3). **P < .01, ***P < .001. F, The presence of circANKRD36 was validated in chondrocytes by RT‐PCR. Divergent primers amplified circANKRD36 from cDNA, but not from genomic DNA. GAPDH was used as a negative control. G, RNA FISH revealed the predominant localization of circANKRD36 in the cytoplasm. circANKRD36 probes were labelled with Cy‐3. Nuclei were stained with DAPI. Scale bar, 50 μm

To investigate the potential role of circANKRD36 in OA, we firstly detected the expression of circANKRD36 in articular cartilage samples from OA or normal patients. As shown in Figure 1A, the expression of circANKRD36 was significantly decreased in tissues from OA patients compared with normal tissues. Moreover, the relative expression of circANKRD36 negatively correlated with modified Mankin Score which we used to evaluate OA severity of the patients according to their pathological gradings (Figure 1B). As IL‐1β has been shown to plan a critical role in the progression of OA by promoting degeneration and apoptosis of chondrocytes, we use IL‐1β treatment to establish in vitro OA cell models. A marked decrease in circANKRD36 expression was found in IL‐1β–treated chondrocytes (Figure 1C). Moreover, percentage of Annexin V–positive cells and inflammatory cytokine (IL‐6 and TNF‐α) both increased in a dose‐dependent manner upon IL‐1β treatment (Figure 1D,E). To rule out the possibility of detecting ANKRD36 liner mRNA along with circANKRD36, we designed convergent primers to amplify ANKRD36 mRNA and divergent primers to amplify circANKRD36 using cDNA and genomic DNA (gDNA). circANKRD36 was amplified by divergent primers in cDNA but not in gDNA (Figure 1F). Furthermore, RNA fluorescence in situ hybridization (FISH) showed that circANKRD36 mainly localized in cytoplasm (Figure 1G). These results indicated that circANKRD36 might be involved in regulation of human OA.

### 3.2 circANKRD36 protects chondrocytes from IL‐1β apoptosis and inflammation

**FIGURE 2:**
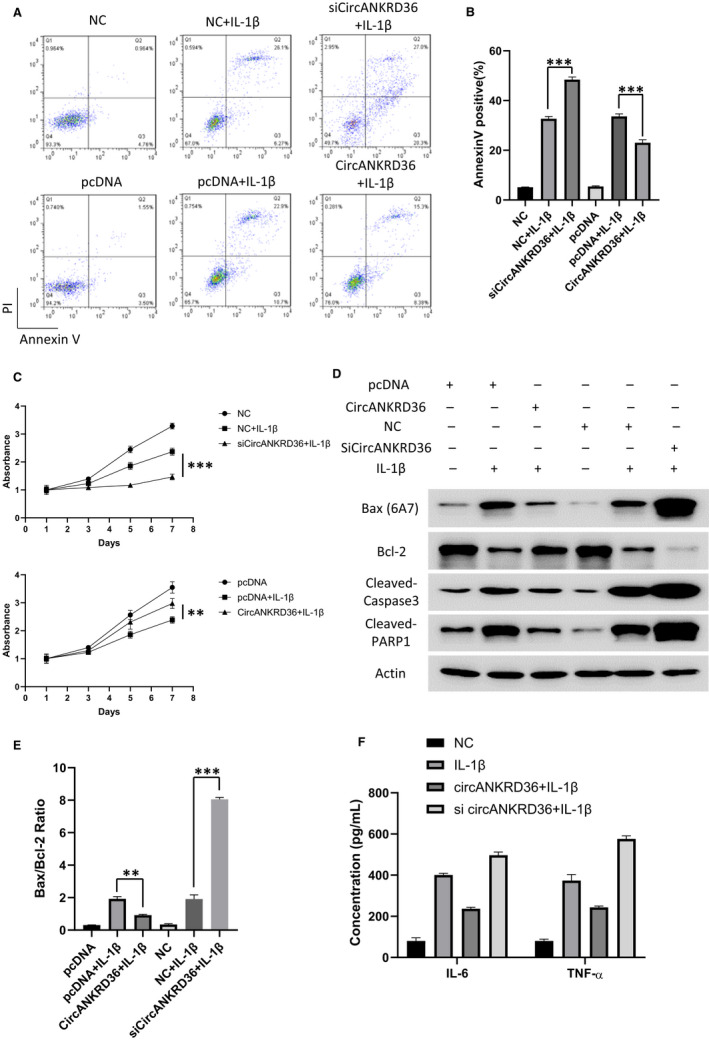
circANKRD36 prevents apoptosis and inflammation in chondrocytes. A, Flow cytometry was conducted to measure the apoptosis rate of human chondrocytes treated as indicated. 1 ng/mL IL‐1β was used to treat the cells. B, Annexin V–positive cell rates in Figure 2Awere statistically analysed. Data are presented as means ± SD (n = 3). **P < .01, ***P < .001. C, Chondrocytes were transfected as indicated and treated with 0.5 ng/mL IL‐1β for different times. Cell viability was measured by CCK‐8 assay. Data represent the means ± SD (n = 3). **P < .01, ***P < .001. D, Apoptosis protein biomarkers Bax(6A7), Bcl‐2, cleaved caspase 3 and PARP1 were measured by Western blot. 1 ng/mL IL‐1β was used to treat the cells. E, The ratio of Bax/Bcl‐2 was calculated. Data are presented as means ± SD (n = 3). **P < .01, ***P < .001. F, Chondrocytes were transfected as indicated and treated with 1 ng/mL IL‐1β. The inflammation cytokine proteins IL‐6 and TNF‐α in culture medium were tested by ELISA Kit

The decreased expression of circANKRD36 in OA tissues indicated that circANKRD36 may function as a suppressor of OA. To test this hypothesis, we performed flow cytometry to measure the apoptosis induced by IL‐1β in chondrocytes with circANKRD36 overexpression or knockdown (Figure 2A). As shown in Figure 2A, the overexpression of circANKRD36 partially rescued the apoptosis induced by IL‐1β, whereas the knockdown of circANKRD36 promoted the apoptotic rate. Statistical analysis of Annexin V–positive cells is shown in Figure 2B. Meanwhile, we performed CCK‐8 assay to investigate the long‐term effect of circANKRD36. Knockdown of circANKRD36 significantly prevented cell proliferation of chondrocytes, whereas the overexpression of circANKRD36 further promoted proliferation in IL‐1β treatment (Figure 2C). In addition, several apoptosis markers, including Bax/Bcl‐2 and cleavage of caspase 3 and PARP1, have been detected by Western blot. Overexpression of circANKRD36 inhibited the elevated Bax/Bcl‐2 ratio and caspase 3/PARP1 cleavage induced by IL‐1β, whereas the knockdown of circANKRD36 caused opposite effect (Figure 2D,E). Furthermore, the pro‐inflammatory cytokines, including IL‐6 and TNF‐α, were detected by ELISA in culture medium of chondrocytes. The inflammation results correlated well with the apoptosis rate induced by IL‐1β (Figure 2F). These results demonstrated that the overexpression of circANKRD36 alleviated apoptosis and inflammation induced by IL‐1β in chondrocytes.

### 3.3 circANKRD36 serves as a sponge for miR‐599 and functions in OA by targeting miR‐599

**FIGURE 3:**
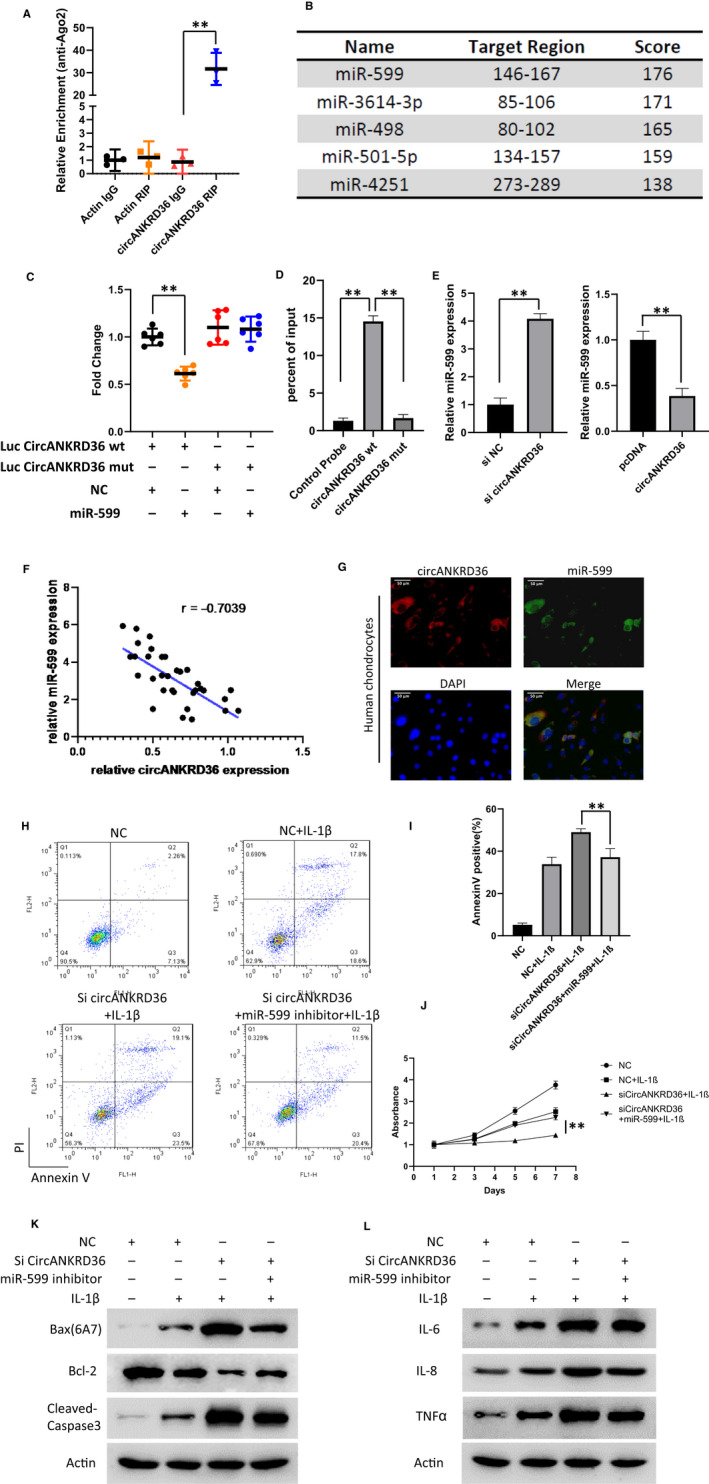
circANKRD36 acts as a miRNA sponge for miR‐599. A, AGO2 IP assay was performed to detect circANKRD36**l**evels in 293T cells stably expressing AGO2. Data are presented as means ± SD (n = 3). **P < .01. B, Bioinformatic analysis of circANKRD36‐binding miRNAs. C, Luciferase activity was measured in circANKRD36 WT, Mut plasmids with miR‐599 mimic and inhibitor cotransfected 293T cells. D, RNA pull‐down was conducted to measure the miR‐599 expression on circ‐ANKRD36 WT and Mut enrichment. Data are presented as means ± SD (n = 3). **P < .01. E, Relative expression of miR‐599 in si‐NC/si‐circANKRD36 and NC/pcDNA‐circANKRD36–infected chondrocytes. Data are presented as means ± SD (n = 3). **P < .01. F, The correlation between circANKRD36 and miR‐599 expression level in OA tissues analysed by Pearson (r = 0.7039,*P* < .05). G, FISH images showing the co‐localization of circANKRD36 and miR‐599 in chondrocytes. miR‐599 probes were labelled with Alexa Fluor 488, whereas circANKRD36 probes were tagged with Cy3. Nuclei were stained with DAPI. Scale bar, 50 μm. H, Flow cytometry was conducted to measure the apoptosis rate of human chondrocytes treated as indicated. 1 ng/mL IL‐1β was used to treat the cells. I, Annexin V–positive cell rates in Figure 3Hwere statistically analysed. Data are presented as means ± SD (n = 3). **P < .01. J, Chondrocytes were transfected as indicated and treated with 1 ng/mL IL‐1β for 48 h. Cell viability was measured by CCK‐8 assay. Data represent the means ± SD (n = 3). **P < .01. K, Apoptosis protein biomarkers Bax(6A7), Bcl‐2, cleaved caspase 3 and PARP1 were measured by Western blot. 1 ng/mL IL‐1β was used to treat the cells. L, The inflammation proteins IL‐6, IL‐8 and TNF‐α were tested by performing Western blot of chondrocytes with indicated treatment

Previous studies have reported that circRNAs can act as miRNA sponges. By sharing one or more microRNA response elements, circRNAs can bind to miRNAs, which in turn adjust the expression of miRNAs. To determine whether circANKRD36 can function as miRNA sponges, we initially searched cirANKRD36‐binding proteins in the Circular RNA Interactome database and found that Argonaute‐2 (Ago2) might bind circANKRD36. To test this hypothesis, we conducted RNA immunoprecipitation (RIP) assay in Flag‐Ago2–transfected cells and found the specific enrichment of endogenous circANKRD36 (Figure 3A). Then, we used bioinformatics software (RegRNA) to predict the potential circRNA/miRNA interactions and found circANKRD36 might interact with five miRNAs (Figure 3B). Among these putative binding miRNAs, miR‐599 has been reported to be involved in apoptosis and inflammatory response. Therefore, miR‐599 was selected for further analysis. Luciferase assay and RNA pull‐down were used to confirm the binding of circANKRD36 to miRNA‐599 (Figure 3C,D). In addition, the expression of miR‐599 was up‐regulated with circANKRD36 knockdown, whereas down‐regulated with circANKRD36 overexpression (Figure 3E). Consistent with this, relative expression of miR‐599 in OA tissues well correlated with the expression of circANKRD36 (Figure 3F). Moreover, FISH experiment further confirmed the co‐localization of circANKRD36 and miR‐599 (Figure 3G). These results indicated that circANKRD36 functions as a sponge for miR‐599.

To examine whether circANKRD36 functions in OA via regulating miR‐599, we cotransfected cells with sh‐circANKRD36 and miR‐599 inhibitor adenovirus. Knockdown of circANKRD36 promoted the apoptosis induced by IL‐1β, and miR‐599 inhibitor blocked this effect (Figure 3H). Statistical analysis of apoptotic rate is shown in Figure 3I. Furthermore, long‐term proliferation activity of cells was tested by CCK‐8 assay and indicated the consistent conclusion (Figure 3J). Western blot of Bax/Bcl‐2 and cleaved caspase 3 confirmed the anti‐apoptotic effect of miR‐599 inhibitor (Figure 3K). Moreover, the pro‐inflammatory cytokine production also showed consistent inflammation result with the apoptosis (Figure 3L).

In conclusion, these results suggest that circANKRD36 acts as a sponge for miR‐599 and protects apoptosis and inflammation by targeting miR‐599.

### 3.4 circANKRD36 promotes Casz1 expression by targeting miR‐599

**FIGURE 4:**
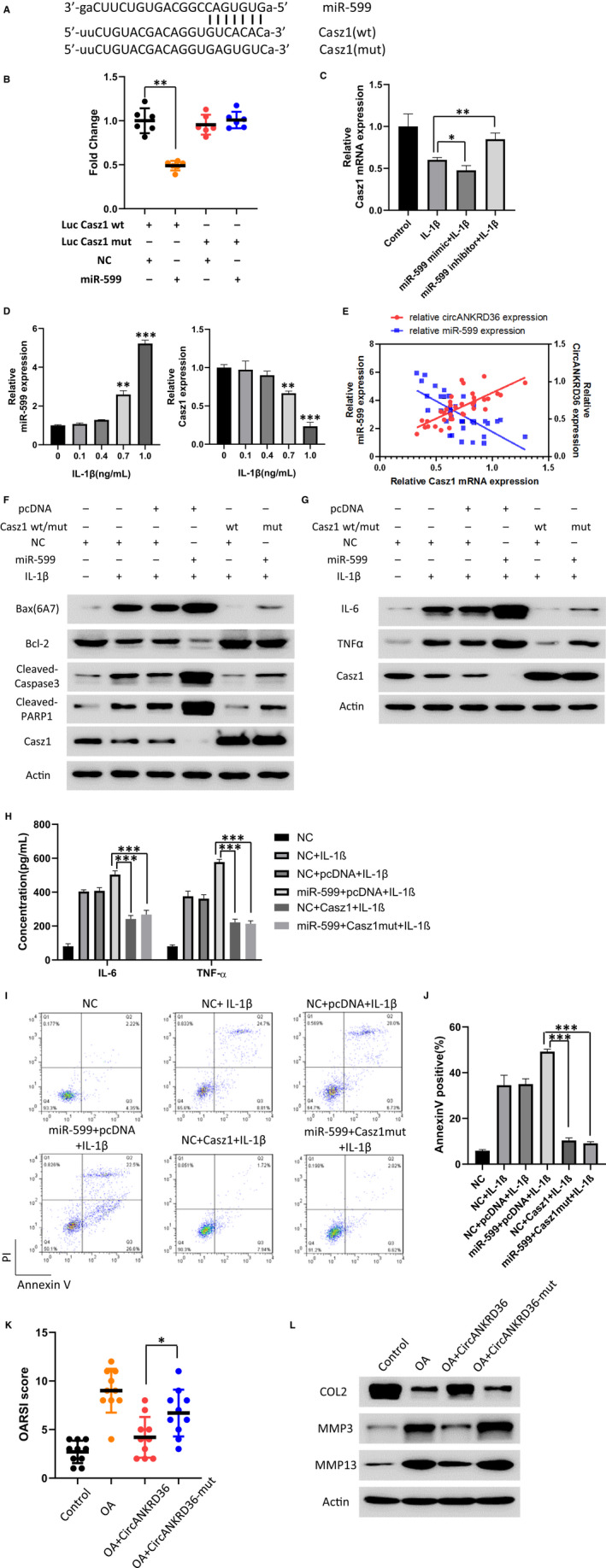
circANKRD36 up‐regulates Casz1 expression by targeting miR‐599. A, Binding sites between miR‐599 and Casz1 are shown. B, Luciferase activity was measured in miR‐599 and Casz1 WT, and Mut cDNA plasmids infected 293T. Data are presented as means ± SD (n = 3). **P < .01. C, Relative expression of Casz1 in human chondrocytes with indicated treatment. Data are presented as means ± SD (n = 3). *P < .05, **P < .01. D, Relative expression of Casz1 and miR‐599 in human chondrocytes with indicated treatment. Data are presented as means ± SD (n = 3). **P < .01, ***P < .001. E, The correlation between miR‐599/Casz1 and circANKRD36/Casz1 expression level in OA tissues analysed by Pearson. F, Apoptosis protein biomarkers Bax(6A7), Bcl‐2, cleaved caspase 3 and PARP1 were measured by Western blot of chondrocytes with indicated treatment. G, The inflammation proteins IL‐6 and TNF‐α were tested by performing Western blot and PCR of chondrocytes with indicated treatment. H, Chondrocytes were transfected as indicated and treated with 1 ng/mL IL‐1β. The inflammation cytokine proteins IL‐6 and TNF‐α in culture medium were tested by ELISA Kit. I, Flow cytometry was conducted to measure the apoptosis rate of human chondrocytes treated as indicated. 1 ng/mL IL‐1β was used to treat the cells. J, Annexin V–positive cell rates in Figure 4Iwere statistically analysed. Data are presented as means ± SD (n = 3). **P < .01. K, ACLT‐induced OA mice were injected with AAV‐negative control, WT AAV circANKRD36 or Mut AAV circANKRD36, and the degree of knee OA was evaluated by OARSI scoring according to the Safranin‐O staining (n = 12). *P < .05. L, Catabolic enzymes (MMP‐3 and MMP‐13) and ECM composition (COL2A1) in mice cartilage tissues were measured by Western blot with indicated treatment

To investigate the potential target of miR‐599, we searched for the putative gene target of miR‐599 via bioinformatic analysis. We found that Casz1, a conserved transcription factor, was a direct target of miR‐599 (Figure 4A). To validate the direct targeting of Casz1 by miR‐599, the wild‐type (wt) Casz1‐targeted sequence or a mutant variant was cloned into a dual‐luciferase reporter vector. The effect of miR‐599 on luciferase activity was detected in 293T cells. As shown in Figure 4B, miR‐599 significantly inhibited luciferase activity of wild‐type Casz1 (Casz1 wt), whereas mutation of the miR‐599‐binding sites (Casz1 mut) abolished the inhibitory effect of miR‐599 (Figure 4B). Overexpression of miR‐599 mimic/inhibitor showed the consistent effect in regulating Casz1 mRNA expression (Figure 4C). Meanwhile, we also detected miR‐599 and Casz1 expression in IL‐1β–treated human chondrocytes. The expression of miR‐599 increased significantly upon IL‐1β treatment, whereas Casz1 mRNA level decreased, both in dose‐dependent manner (Figure 4D). In addition, we analysed the expression of miR‐599/Casz1 and circANKRD36/Casz1 in OA tissues and found the consistent correlation of both by Pearson (Figure 4E). These results showed that Casz1 is a direct target of miR‐599.

To determine the function of Casz1 in miR‐599‐promoted apoptosis, we co‐infected chondrocytes with miR‐599 and Casz1 expressing lentivirus and detected apoptotic rate of chondrocytes by flow cytometry and Western blot (Figure 4F,I,J). miR‐599 promoted IL‐1β–induced apoptosis, whereas the overexpression of Casz1 mut, which is not regulated by miR‐599, rescued this effect. Consistently, the pro‐inflammatory cytokines IL‐6, IL‐8 and TNF‐α were blocked by Casz1 overexpression (Figure 4G,H).

In order to investigate the role of circANKRD36 in vivo, WT or Mut adenovirus (AAV), circANKRD36 was injected to anterior cruciate ligament transection (ACLT)‐induced OA mice. Quantitative analysis with Osteoarthritis Research Society International (OARSI) scoring showed that WT AAV circANKRD36 markedly lowered OARSI scores, whereas Mut AAV circANKRD36 treatment showed no function (Figure 4I). The injection of WT AAV circANKRD36 alleviated the degeneration changes in the cartilage tissue, such as increased ECM composition (COL2, MMP3, MMP13) in the OA mouse model (Figure 4J). Taken together, these results demonstrated that circANKRD36 promotes Casz1 expression by targeting miR‐599, thus protects chondrocyte apoptosis and inflammation.

## 4 DISCUSSION

Up to now, there are still no effective therapeutic drugs approved for clinical treatment of OA.^2^, ^4^, ^16^ To develop effective therapeutics, it is crucial to investigate the underlying mechanisms of OA. OA involves multiple pro‐inflammatory cytokines, including TNF, IL‐6 and IL‐8, which are secreted by chondrocytes triggered along with apoptosis.^1^, ^4^ Thus, human chondrocytes treated by IL‐1β have always been used to establish an inflammatory damage model to study OA in vitro.^4^

In this study, we used IL‐1β–treated chondrocytes to study the molecular mechanism of OA and found circANKRD36 as a novel suppressor of chondrocyte apoptosis and inflammation. As newly discovered non‐coding RNAs, circRNAs have been reported to take part in the pathogenesis of OA disease.^17^ Several circRNAs, such as circSERPINE2 and circRNA‐CDR1as, were found to function as miRNA sponges to regulate chondrocyte proliferation, apoptosis and inflammation.^17^, ^18^, ^19^ circANKRD36 was recently discovered in diabetes and was reported to play an anti‐inflammatory damage efficacy.^11^ However, it has been reported that circANKRD36 serves as a pro‐inflammatory factor in LPS‐treated HaCaT cells, which were performed as a bedsore model.^20^ The dichotomous effect of circANKRD36 may arise with different signalling pathways in specific tissues.

Casz1 orchestrates cell specification and differentiation in many cell lineages, including neuroblasts, cardiomyocytes and lymphoid cells.^13^, ^15^ The function of Casz1 in cartilage chondrocytes has not been studied before. Our research also found a novel regulatory pathway of Casz1. It has been reported that Casz1 limits repressive histone marks and enables acquisition of permissive histone marks at several Th17 differentiation gene loci.^13^ Considering its function as a transcriptional factor, we speculate that Casz1 may regulate apoptosis and inflammation by regulating the expression of important cell death and inflammation‐related genes. However, it still remains unknown about the downstream effectors of Casz1 in OA regulation.

miR‐599 has been reported to have dichotomous roles in different cell types.^21^, ^22^ miR‐599 promotes apoptosis in papillary thyroid carcinoma cells by targeting Hey2, which is a transcription factor involved in cell fate and forming boundary.^21^ In our study, miR‐599 is proved to inhibit chondrocyte apoptosis by targeting Casz1, which is also a transcription factor. Considering this, we speculate that miR‐599 may have to function to regulate different pathways by targeting various transcription factors.

In conclusion, we have identified the circANKRD36‐miR‐599‐Casz1 axis as a novel target for the prevention and treatment of OA. cirANKRD36 and Casz1 overexpression may remit the apoptosis and inflammation in chondrocytes upon IL‐1β treatment. Besides, miR‐599 inhibition may also serve as an effective therapeutic strategy for OA treatment.

## CONFLICT OF INTEREST

The authors have declared that no competing interest exists.

## AUTHOR CONTRIBUTION

**Jian‐Lin Zhou:** Conceptualization (equal); Investigation (equal); Resources (equal); Software (equal); Writing‐original draft (equal). **Shuang Deng:** Data curation (equal); Resources (equal); Validation (equal); Writing‐original draft (equal). **Hong‐Song Fang:** Conceptualization (equal); Data curation (equal); Formal analysis (equal); Writing‐original draft (equal). **Xian‐jin Du:** Conceptualization (equal); Formal analysis (equal); Writing‐original draft (equal); Writing‐review & editing (equal). **Hao Peng:** Investigation (equal); Resources (equal); Validation (equal); Writing‐original draft (equal). **qiongjie hu:** Conceptualization (supporting).

## Data Availability

All data generated or analysed during this study are included in this article. Further details are available on request.
